# Supplementary material for: Epigenetic memory of radiotherapy in dermal fibroblasts impairs wound repair capacity in cancer survivors
Source: Nat Commun. 2024 Oct 28;15:9286. doi: 10.1038/s41467-024-53295-1 (PMC11519383; doi:10.1038/s41467-024-53295-1)
Supplement: Supplementary file 1 — Supplementary Information [file 41467_2024_53295_MOESM1_ESM.pdf]

**Supplementary information**

# Epigenetic memory of radiotherapy in dermal fibroblasts impairs wound repair capacity in cancer survivors

Xiaowei Bian[1, †], Minna Piipponen[1, †], Zhuang Liu[1], Lihua Luo[1], Jennifer Geara[1], Yongjian Chen[1], Traimate Sangsuwan[2], Monica Maselli[1], Candice Diaz[3,4], Connor A Bain[5], Evelien Eenjes[6], Maria Genander[6], Michael Crichton[5], Jenna L Cash[7], Louis Archambault[8], Siamak Haghdoost[2,9], Julie Fradette[3,4,10], Pehr Sommar[11,12], Martin Halle[11,12,#], Ning Xu Landén[1,#]

[1]Dermatology and Venereology Division, Department of Medicine Solna, Center for Molecular Medicine, Karolinska Institutet; Stockholm, Sweden

[2]Department of Molecular Biosciences, The Wenner-Gren Institute, Stockholm University; Stockholm, Sweden

[3]Centre de recherche en organogénèse expérimentale de l'Université Laval / LOEX, Québec, QC, Canada

[4]Division of Regenerative Medicine, CHU de Québec-Université Laval Research Centre, Québec, QC, Canada.

[5]Institute of Mechanical, Process and Energy Engineering, School of Engineering and Physical Sciences, Heriot-Watt University, Edinburgh, EH14 4AS, UK

[6]Department of Cell and Molecular Biology, Karolinska Institutet, Solna, Sweden

[7]Centre for Inflammation Research, Institute for Regeneration and Repair, 4-5 Little France Drive, University of Edinburgh, Edinburgh, EH16 4UU

[8]Department of Physics, Université Laval/Centre de Recherche sur le Cancer, Université Laval/Centre de recherche du CHU de Québec, QC, Canada

[9]ABTE/ToxEMAC laboratory, University of Caen Normandy, Advanced Resource Center for HADrontherapy in Europe (ARCHADE), Caen, France

[10]Department of Surgery, Faculty of Medicine, Université Laval, Québec, QC, Canada.

[11]Department of Plastic and Reconstructive Surgery, Karolinska University Hospital, Stockholm, Sweden

[12]Department of Molecular Medicine and Surgery, Karolinska Institutet, Stockholm, Sweden

[†]These authors contributed equally: Xiaowei Bian, Minna Piipponen

[#]These authors jointly supervised this work: Martin Halle, Ning Xu Landén

*Correspondence to Ning Xu Landén, ning.xu@ki.se and Martin Halle, martin.halle@regionstockholm.se

## Supplementary figures

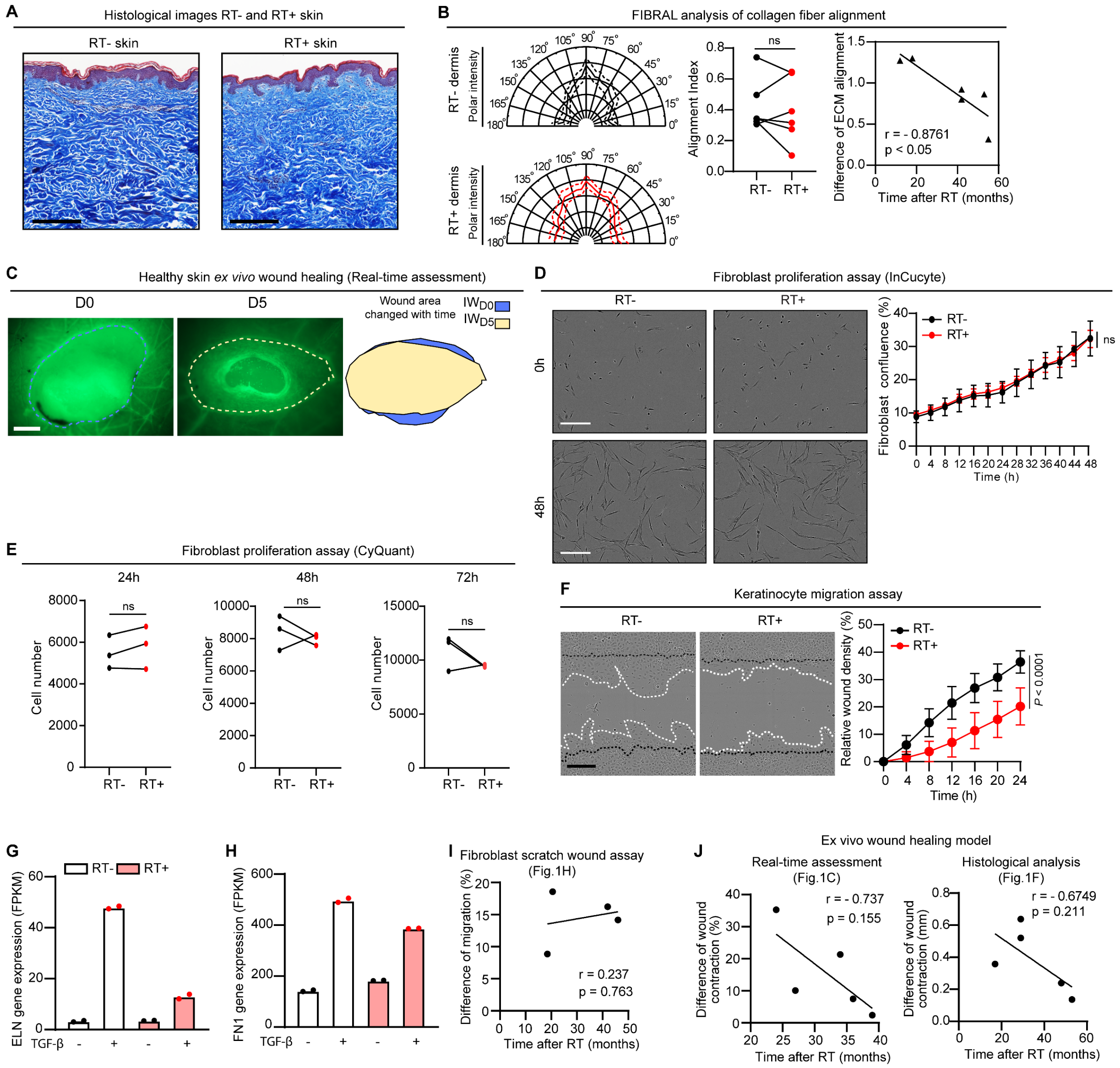


**Supplementary figure 1. Clinical, histological, and cellular functional differences between RT⁻ and RT⁺ skin. (A)** Masson's trichrome staining of human RT⁻ and RT⁺ skin. Scale bar: 250 µm. **(B)** FIBRAL analysis of human RT⁻ and RT⁺ skin: polar plots illustrating collagen fiber alignment (left); comparison of collagen fiber alignment index (middle); correlation analysis of the variation in collagen alignment between RT⁺ and RT⁻ samples with the length of time post-radiotherapy (*n* = 6 donors). **(C)** Representative images of *ex vivo* wounds on healthy skin on day 0 and day 5 post-injury (HS, *n* = 5). The initial wound edges were demarcated with dashed lines. The areas within the initial wound edge ($IW_{time\ point}$) are illustrated (right). Scale bar: 500 µm. **(D)** Representative images and quantification of fibroblast proliferation measured by InCucyte live-

cell imaging system (*n* = 6). Scale bar: 300 µm. **(E)** Quantification of fibroblast proliferation assay using CyQuant assay (*n* = 3 donors). **(F)** Representative images and quantification of keratinocyte scratch wound assay (*n* = 6). Scale bar: 300 µm. RNA-seq of *ELN* **(G)** or *FN1* **(H)** in human $RT^-$ or $RT^+$ fibroblasts treated with or not with TGF-β (*n* = 2). FPKM: Fragments per kilobase of transcript per million mapped reads. Correlation analysis of $RT^+$ vs. $RT^-$ differences of fibroblast migration rate (%) (*n* = 4 donors) **(I)**, and wound contraction rate (%) (*n* = 5 donors) **(J)** with the length of time post-radiotherapy. Data are presented as means ± SD **(D, F)**. Paired two-tailed student's t-test **(B, E)**, two-way ANOVA **(D, F)**, and Pearson's correlation test (**B, I, J**). ns: not significant (P > 0.05). Source data are provided as a Source Data file.

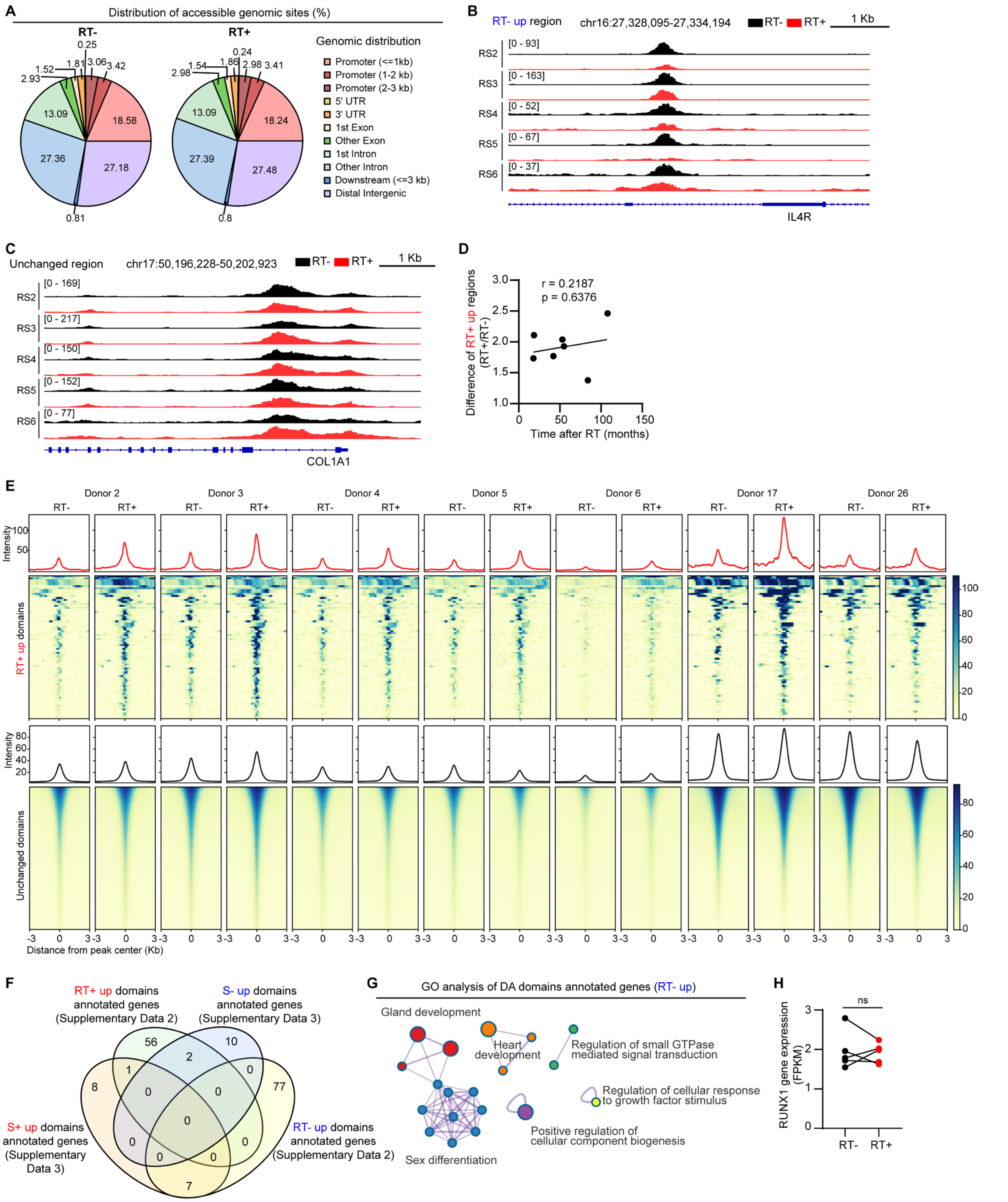


**Supplementary figure 2. Chromatin accessibility changes in RT$^{-}$ and RT$^{+}$ fibroblasts. (A)** Distribution of accessible genomic loci in the RT$^{-}$ (left) and RT$^{+}$ (right) fibroblasts (n = 5 paired samples). Representative integrative genomics viewer (IGV) images of ATAC signals of a RT$^{-}$ up domain on *IL4R* gene **(B)** and an unchanged domain on *COL1A1* gene **(C)**. **(D)** Correlation analysis of the RT$^{+}$ vs. RT$^{-}$ difference of the RT$^{+}$ up domains with the length of time post-radiotherapy (*n*

= 7 donors). **(E)** Heatmap of ATAC-seq signal intensity of the regions with more accessibility in $RT^+$ fibroblasts (upper panel) or the unchanged regions (lower panel). **(F)** Comparison of genes with $RT^-$ up or $RT^+$ up domains against genes exhibiting chromatin accessibility in S- up or S+ up domains. **(G)** GO analysis of the genes with reduced chromatin accessibility in $RT^+$ fibroblasts: each node represents a biological process, and node size indicates the number of genes enriched in that process. **(H)** RNA-seq analysis of RUNX1 expression in $RT^-$ and $RT^+$ fibroblasts ($n$ = 5 donors). Pearson's correlation test **(D)**, or paired two-tailed student's t-test **(H)**. ns: not significant (P > 0.05). Source data are provided as a Source Data file.

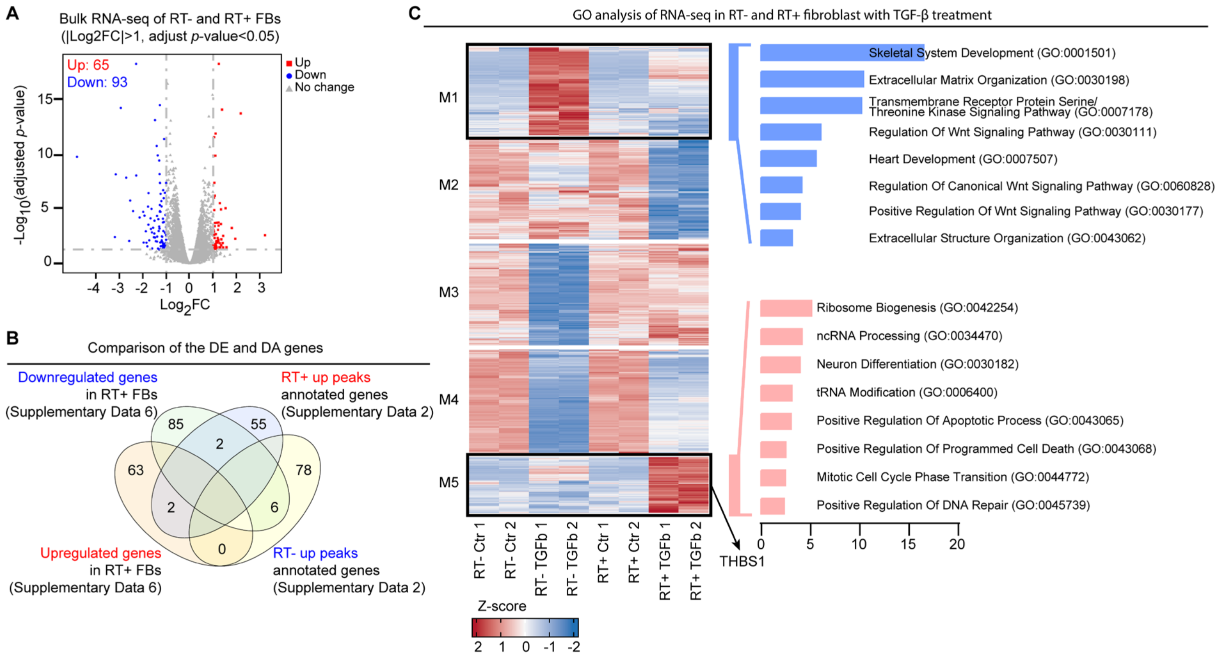


**Supplementary figure 3. Differential gene expression in RT⁻ and RT⁺ fibroblasts. (A)** Volcano plot showing differentially expressed (DE) genes in $RT^+$ vs. $RT^-$ fibroblasts revealed by bulk RNA-seq ($n$ = 5 donors). **(B)** Venn diagram comparing DE genes to DA peaks annotated genes in $RT^+$ vs. $RT^-$ fibroblasts. **(C)** Heatmap of DE genes (ANOVA, P < 0.05) in $RT^+$ and $RT^-$ fibroblasts treated with or not with TGF-β1 for 24 hours, as shown by RNA-seq (left). GO analysis of the genes with expression change patterns M1 and M5 (right, hypergeometric test for enrichment analysis, P < 0.05).

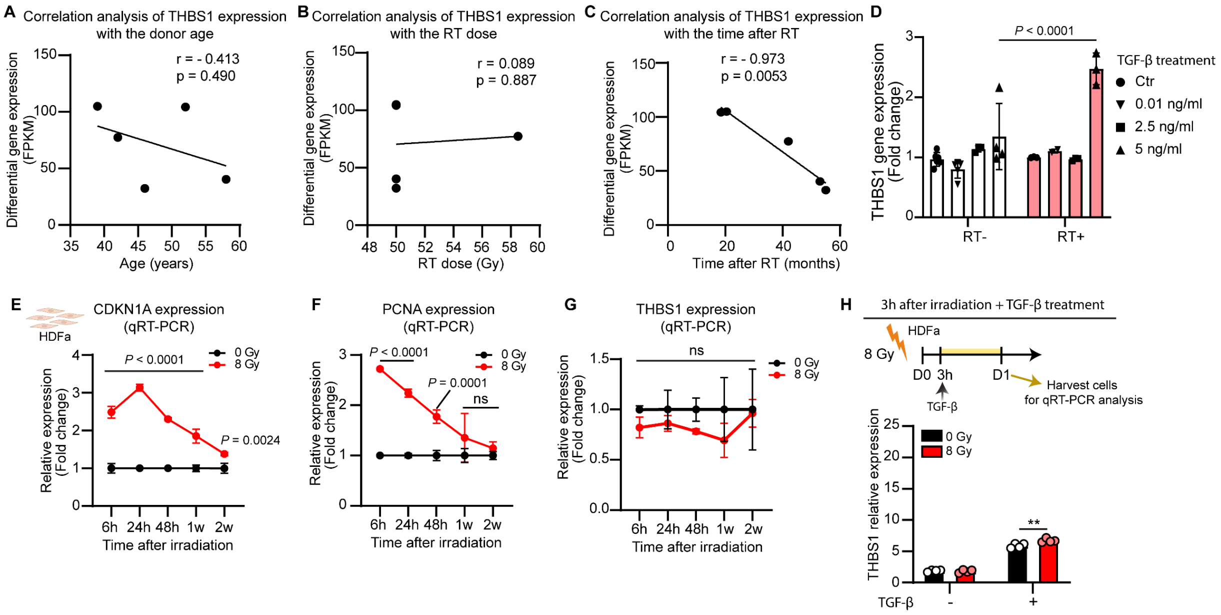


**Supplementary figure 4. *THBS1* expression analysis in RT$^{+}$ and RT$^{-}$ fibroblasts.** Correlation analysis of differential *THBS1* expression in RT$^{+}$ and RT$^{-}$ fibroblasts with patient's age **(A)**, the dose of radiotherapy **(B)**, and the length of time after radiotherapy **(C)** (*n* = 5 donors). **(D)** qRT-PCR of *THBS1* expression in RT$^{-}$ and RT$^{+}$ fibroblasts treated with different doses of TGF-β (RT- Ctr, *n* = 6; RT- 0.01 ng/ml, *n* = 4; other groups *n* = 3). Fibroblasts from healthy donors were irradiated and harvested 6 hours - 2 weeks post-irradiation. qRT-PCR analysis of *CDKN1A* **(E)**, *PCNA* **(F)**, and *THBS1* **(G)** in non-irradiated vs. irradiated fibroblasts (0 Gy, 1w, *n* = 4; other groups *n* = 3). **(H)** Fibroblasts were irradiated and then treated with or not with TGF-β three hours post-irradiation. *THBS1* expression was analyzed by qRT-PCR (*n* = 4). Data are presented as means ± SD **(D-H)**. Pearson's correlation test **(A-C)**, or two-way ANOVA **(D-H)**. ns: not significant (P > 0.05). Source data are provided as a Source Data file.

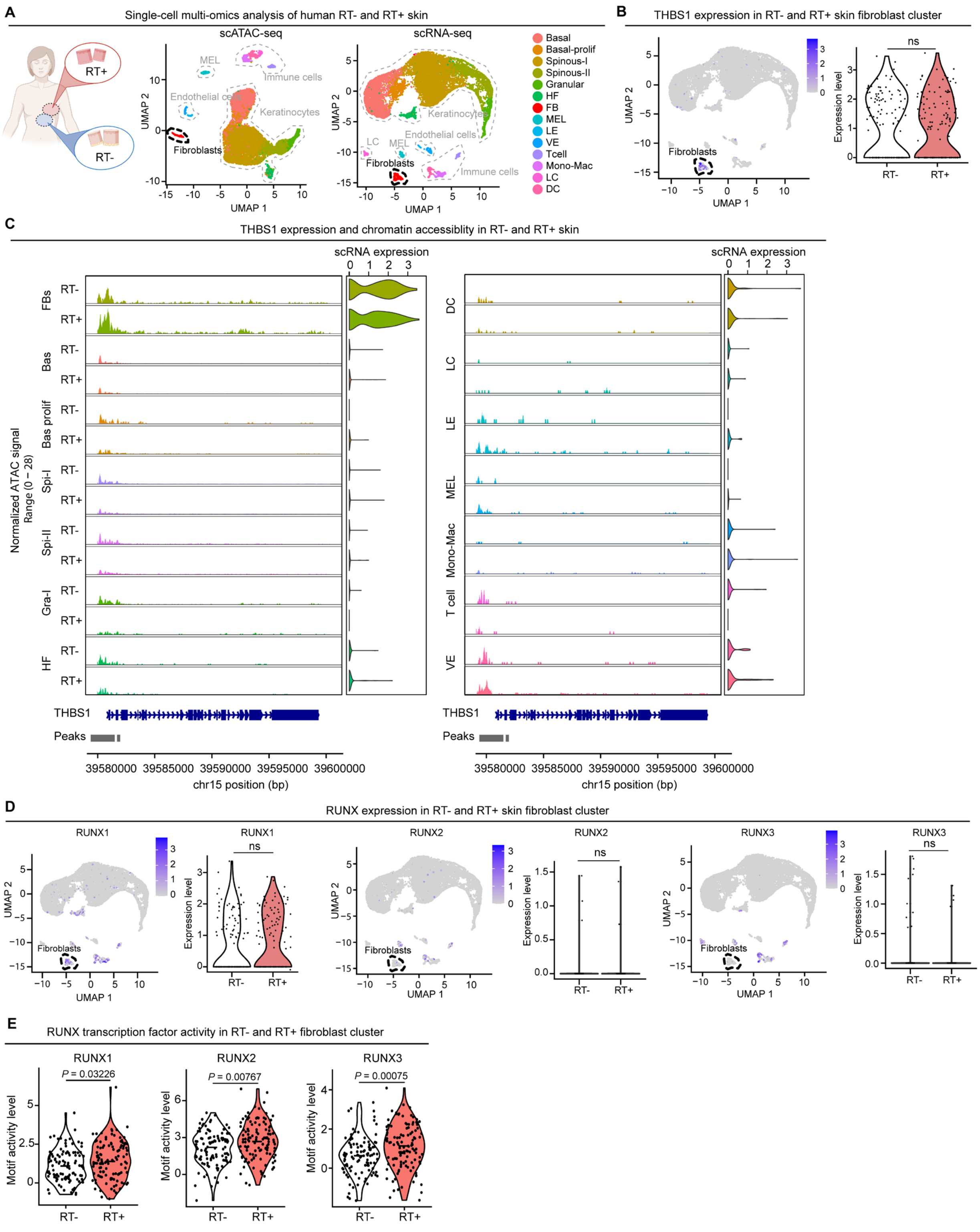


**Supplementary figure 5. Single-cell multiome ATAC + gene expression analysis of RT- and RT+ skin. (A)** Single-cell ATAC-seq (middle) and single-cell RNA-seq (right) UMAP plots of 14,047 cells from RT- and RT+ skin of one donor, color-coded by cell type. **(B)** Single-cell RNA-

seq UMAP plot showing *THBS1* gene expression (left). Violin plot of *THBS1* gene expression in the fibroblast cluster (right). **(C)** Coverage plots showing the genomic track of the *THBS1* gene in each cell cluster shown by single-cell ATAC-seq. **(D)** Single-cell RNA-seq UMAP plots showing *RUNX1*, *RUNX2*, and *RUNX3* gene expression. Violin plots of *RUNX1, RUNX2*, and *RUNX3* gene expression in the fibroblast cluster. **(E)** RUNX transcription factor activity in $RT^-$ and $RT^+$ fibroblasts. Mann-Whitney U test **(B, D, E)**. ns: not significant ($P > 0.05$).

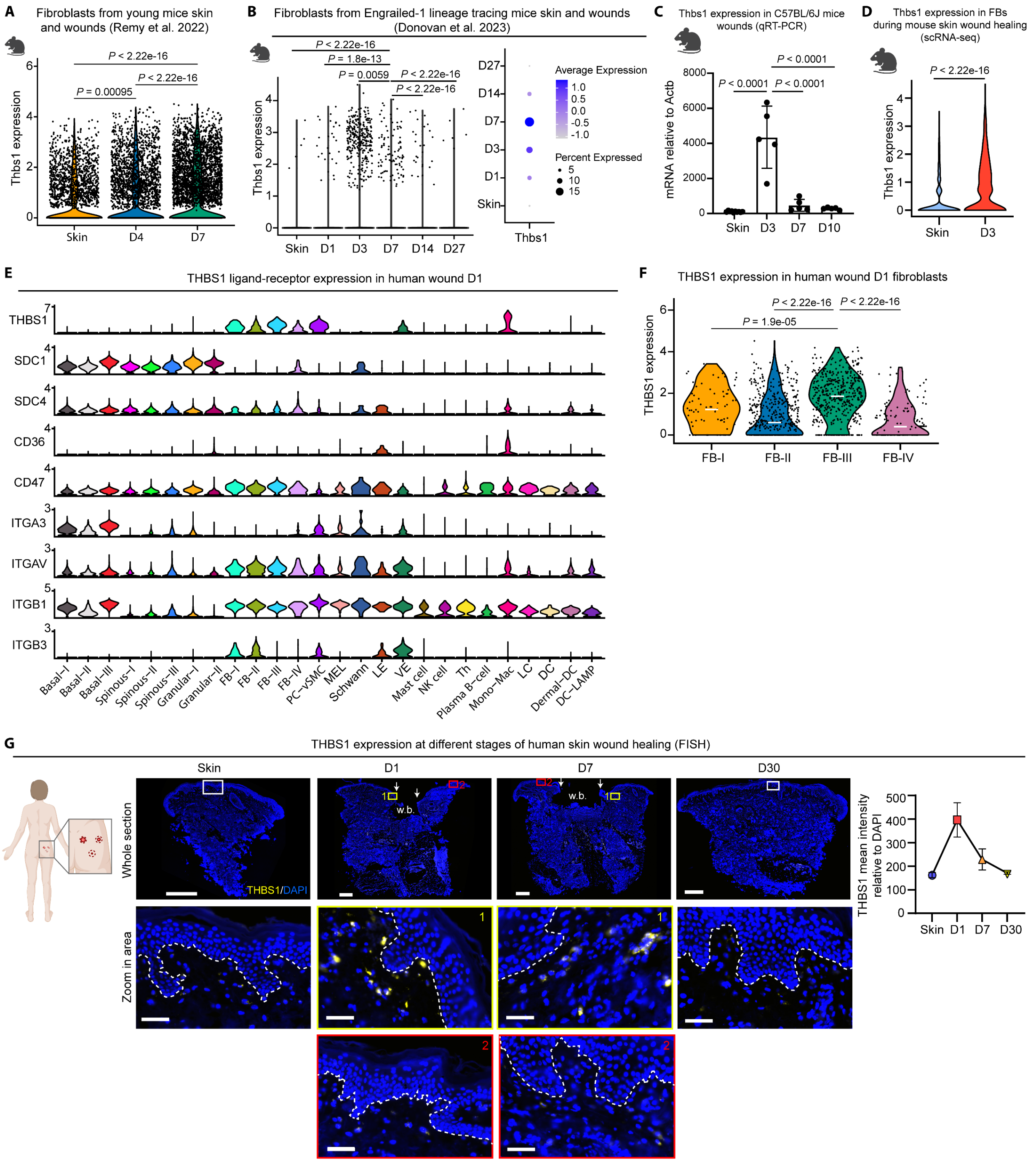


**Supplementary figure 6. *THBS1* expression in mouse and human skin wound healing. (A, B)** *Thbs1* expression patterns in murine skin and wound fibroblasts, as revealed by published single-cell RNA-seq datasets. **(C)** qRT-PCR analysis of *Thbs1* in the skin and Day 3, 7, and 10 wounds of C57BL/6 mice (Skin, $n$ = 10; D3, D7, D10, $n$ = 5). **(D)** *Thbs1* expression in fibroblasts from the skin and Day 3 wounds of C57BL/6 mice analyzed by single-cell RNA-seq. **(E)** *THBS1* and its receptor expression in each cell type in human Day 1 acute wounds, as shown by single-cell RNA-

seq analysis. **(F)** *THBS1* expression in fibroblast sub-clusters in human Day 1 acute wounds. **(G)** Representative FISH images of *THBS1* expression in human skin, Day 1, Day 7, and Day 30 acute wounds (Skin and Wound D7, n =3; Wound D1, n = 4; Wound D30, n =5). Dashed lines indicate the epidermal-dermal junctions. w.b.: wound bed. Rectangles indicate the zoom-in areas. The yellow rectangles labeled with '1' are at the wound edges. The red rectangles marked with '2' are further from the wound edges. Scale bars: 500 µm (upper panel) and 50 µm (middle and lower panels). Mann-Whitney U test **(A, B, D, F)**, one-way ANOVA **(C)**. Source data are provided as a Source Data file.

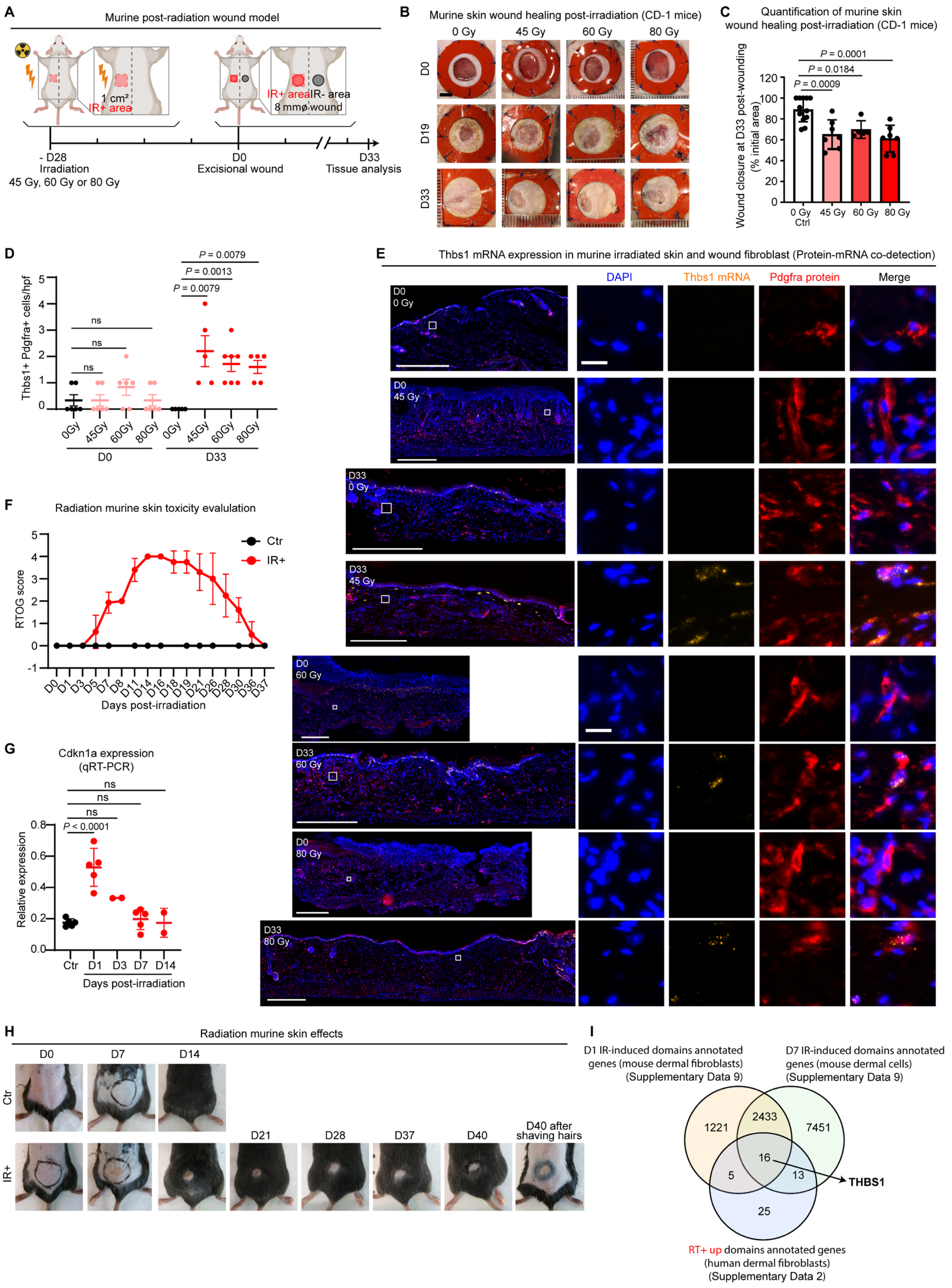
A
Murine post-radiation wound model
1 cm²
IR+ area
IR+ area
IR- area
8 mmø wound
- D28
Irradiation
45 Gy, 60 Gy or 80 Gy
D0
Excisional wound
D33
Tissue analysis
B
Murine skin wound healing post-irradiation (CD-1 mice)
0 Gy
45 Gy
60 Gy
80 Gy
D0
D19
D33
C
Quantification of murine skin wound healing post-irradiation (CD-1 mice)
Wound closure at D33 post-wounding (% initial area)
P = 0.0001
P = 0.0184
P = 0.0009
0 Gy Ctrl
45 Gy
60 Gy
80 Gy
D
Thbs1+ Pdgfra+ cells/hpf
P = 0.0079
P = 0.0013
P = 0.0079
ns
ns
ns
0Gy
45Gy
60Gy
80Gy
D0
D33
E
Thbs1 mRNA expression in murine irradiated skin and wound fibroblast (Protein-mRNA co-detection)
DAPI
Thbs1 mRNA
Pdgfra protein
Merge
D0 0 Gy
D0 45 Gy
D33 0 Gy
D33 45 Gy
D0 60 Gy
D33 60 Gy
D0 80 Gy
D33 80 Gy
F
Radiation murine skin toxicity evalulation
RTOG score
Ctr
IR+
D0 D1 D3 D5 D7 D8 D11 D14 D16 D18 D19 D21 D26 D28 D30 D36 D37
Days post-irradiation
G
Cdkn1a expression (qRT-PCR)
Relative expression
P < 0.0001
ns
ns
ns
Ctr
D1
D3
D7
D14
Days post-irradiation
H
Radiation murine skin effects
D0
D7
D14
D21
D28
D37
D40
D40 after shaving hairs
Ctr
IR+
I
D1 IR-induced domains annotated genes (mouse dermal fibroblasts) (Supplementary Data 9)
D7 IR-induced domains annotated genes (mouse dermal cells) (Supplementary Data 9)
1221
2433
7451
16
5
13
25
THBS1
RT+ up domains annotated genes (human dermal fibroblasts) (Supplementary Data 2)

**Supplementary figure 7. Murine skin post-irradiation wound healing models. (A)** Illustration of the murine post-irradiation wound model (CD-1 mice). **(B)** Representative images of murine skin wound closure post-irradiation (CD-1 mice) (0 Gy, n = 12; 45 Gy and 80 Gy, n = 7; 60 Gy, n = 5). Scale bar: 4 mm. **(C)** Quantification of CD-1 mice wound healing at day 33 post-wounding in the non-irradiated (0 Gy, Ctrl) and irradiated (45 Gy, 60 Gy, and 80 Gy) murine skin (0 Gy, *n* = 12; 45 Gy and 80 Gy, *n* = 7; 60 Gy, *n* = 5). **(D)** Quantification of $Thbs1^+$ $Pdgfra^+$ fibroblasts per high-power field (hpf) in CD-1 mice wound healing (D0, *n* = 6; 0 Gy, 45 Gy, and 80 Gy at D33, *n* = 5; 60 Gy at D33, *n* = 7). **(E)** Representative images of co-staining of Thbs1 mRNA and Pdgfra protein in non-irradiated (0 Gy) and irradiated (45 Gy, 60 Gy, and 80Gy) CD-1 mice murine skin (D0) and 33 days (D33) post-wounding (D0, *n* = 6; 0 Gy, 45 Gy, and 80 Gy at D33, *n* = 5; 60 Gy at D33, *n* = 7). **(F)** Evaluation of irradiation-induced murine skin toxicity using the RTOG score (Ctr, *n* = 4; $IR^+$, *n* = 18) (C57BL/6 mice). Scale bars: 500 µm or 20 µm in the zoom-in area. **(G)** qRT-PCR analysis of *Cdkn1a* at day 1, 3, 7, and 14 post-irradiation in the non-irradiated control mice (Ctr), and irradiated ($IR^+$) murine skin dermal cells (Ctr, *n* = 5; $IR^+$, *n* = 14) (C57BL/6 mice). **(H)** Representative images of radiation-induced murine skin effects (C57BL/6 mice) (Ctr, *n* = 4; $IR^+$, *n* = 18). **(I)** Comparison of genes with $RT^+$ up domains versus genes induced by irradiation in C57BL/6 murine skin dermal cells. The overlapping genes are detailed in Supplementary Data 9. Data are presented as means ± SD. One-way ANOVA **(C, G)**, two-tailed Mann-Whitney U test **(D)**. ns: not significant. Supplementary figure 7A was created with BioRender.com released under a Creative Commons Attribution-NonCommercial-NoDerivs 4.0 International license. Source data are provided as a Source Data file.

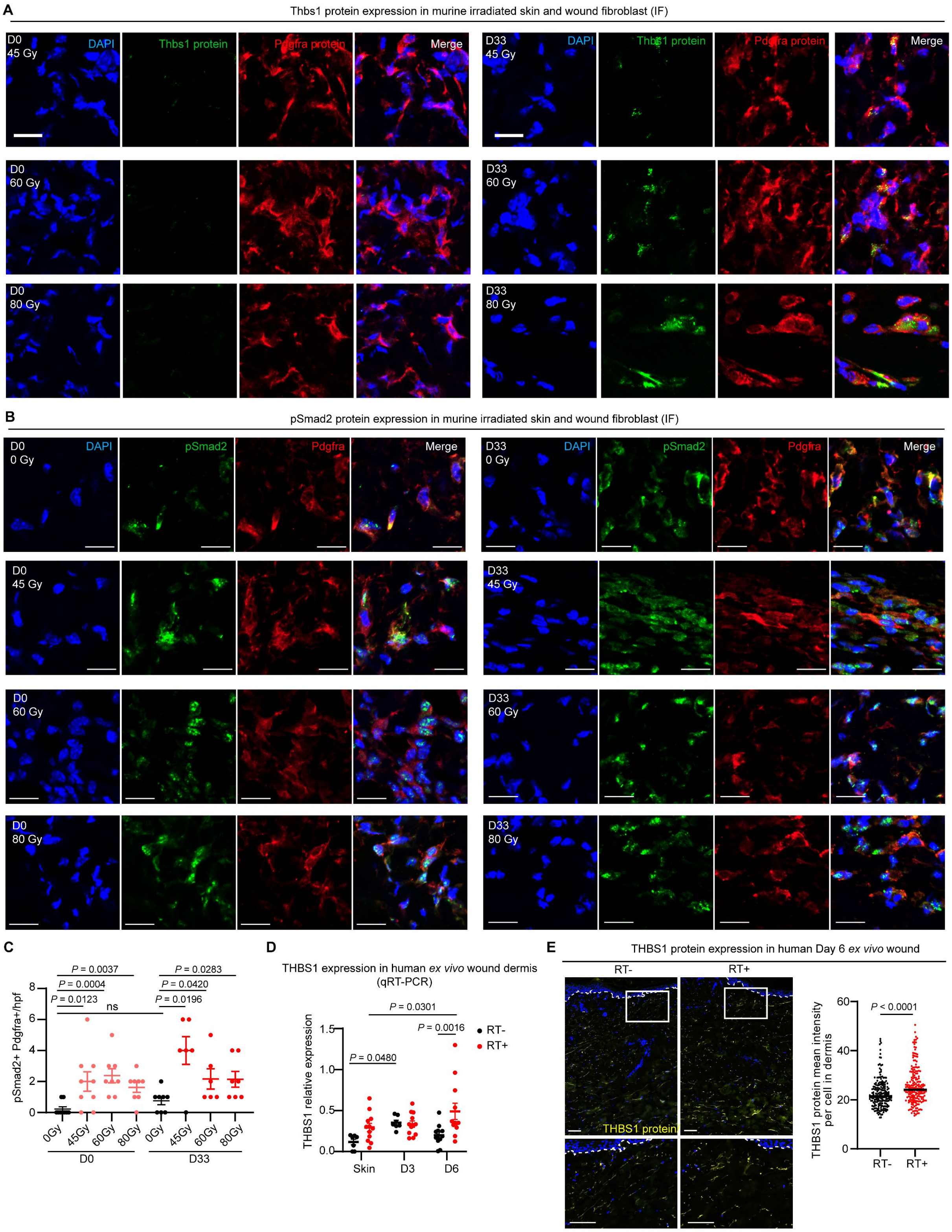


**Supplementary figure 8. THBS1 expression in wounds of irradiated skin. (A)** Representative IF images of co-staining of Thbs1 and Pdgfra proteins in irradiated (45, 60, or 80 Gy) CD-1 murine skin 0- and 33-days post-wounding. The process of producing these representative images was

repeated (0 Gy, *n* = 12; 45 Gy and 80 Gy, *n* = 7; 60 Gy, *n* = 5). Scale bars: 20 µm. **(B)** Representative IF staining images of pSmad2 and Pdgfra proteins in non-irradiated (0 Gy) and irradiated (45, 60, or 80 Gy) CD-1 murine skin D0 and D33 post-wounding (0 Gy and 45 Gy, D0 *n* = 9; 60 Gy and 80 Gy, D0 *n* = 8; 0 Gy, D33 *n* = 8; 45 Gy and 60 Gy, D33 *n* = 6; 80 Gy, D33 *n* = 7). Scale bars: 20 µm. **(C)** Quantification of $pSmad2^{+}Pdgfra^{+}$ fibroblasts per high-power field (hpf) (0 Gy and 45 Gy, D0 *n* = 9; 60 Gy and 80 Gy, D0 *n* = 8; 0 Gy, D33 *n* = 8; 45 Gy and 60 Gy, D33 *n* = 6; 80 Gy, D33 *n* = 7). **(D)** qRT-PCR analysis of dermal THBS1 expression in human $RT^{-}$ versus $RT^{+}$ *ex vivo* wounds at 0, 3, and 6 days post-wounding (RT- Skin and D3, *n* = 7; other groups, *n* = 12). **(E)** Representative IF images and quantification of THBS1 protein expression intensity in $RT^{-}$ versus $RT^{+}$ human *ex vivo* wounds (*n* = 195) 6 days post-wounding. Each dot in the quantification plot represents the THBS1 signal intensity per dermal cell. Scale bars: 100 µm (upper panel) and 50 µm (lower panel). Data are presented as means ± SD. Mann-Whitney U test **(C**, **E)**, two-way ANOVA **(D)**. ns, not significant ($P > 0.05$). Source data are provided as a Source Data file.

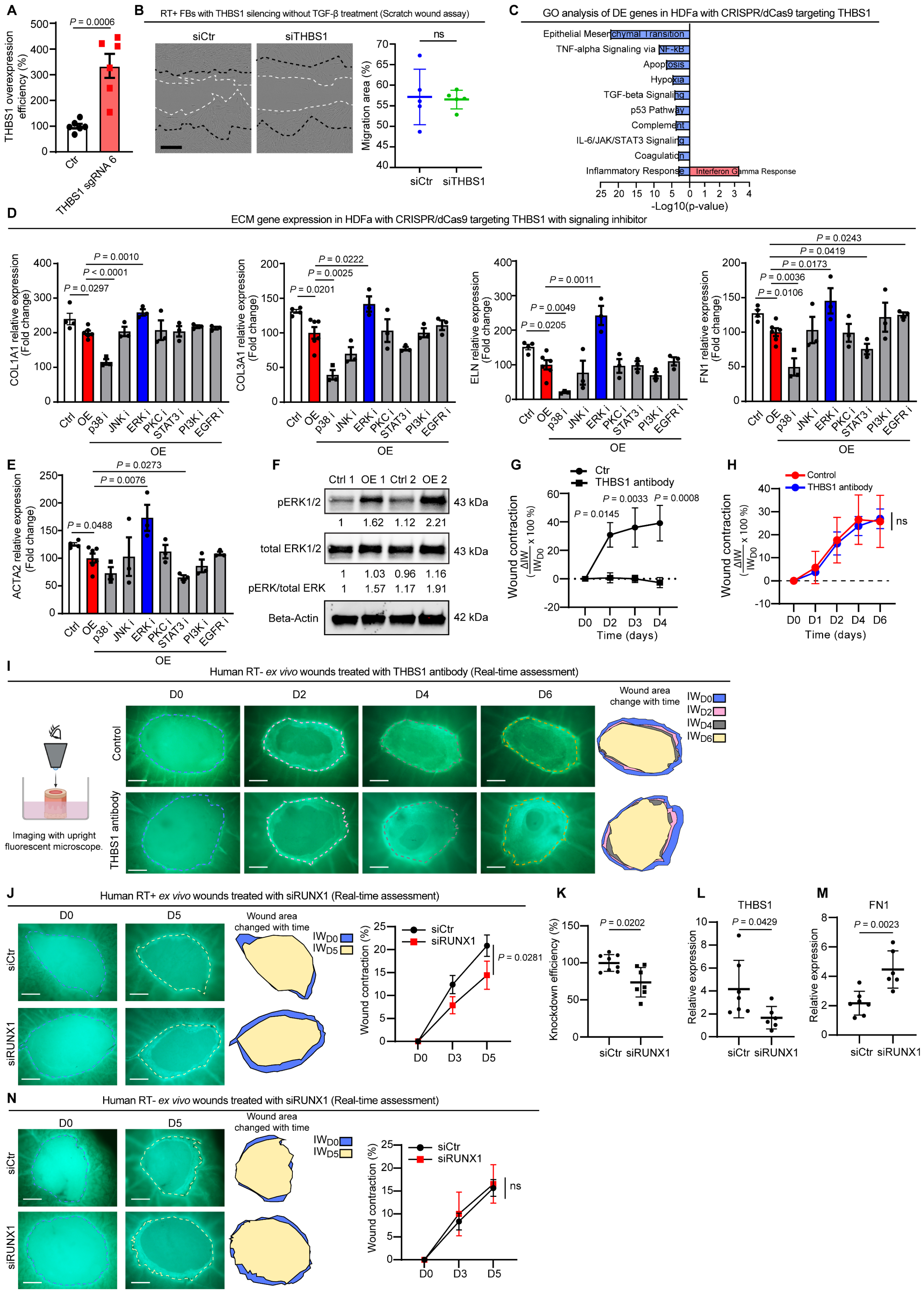

A
THBS1 overexpression efficiency (%)
P = 0.0006
Ctr
THBS1 sgRNA 6
B
RT+ FBs with THBS1 silencing without TGF-β treatment (Scratch wound assay)
siCtr
siTHBS1
Migration area (%)
ns
C
GO analysis of DE genes in HDFa with CRISPR/dCas9 targeting THBS1
Epithelial Mesenchymal Transition
TNF-alpha Signaling via NF-kB
Apoptosis
Hypoxia
TGF-beta Signaling
p53 Pathway
Complement
IL-6/JAK/STAT3 Signaling
Coagulation
Inflammatory Response
Interferon Gamma Response
-Log10(p-value)
D
ECM gene expression in HDFa with CRISPR/dCas9 targeting THBS1 with signaling inhibitor
COL1A1 relative expression (Fold change)
COL3A1 relative expression (Fold change)
ELN relative expression (Fold change)
FN1 relative expression (Fold change)
Ctrl OE p38 i JNK i ERK i PKC i STAT3 i PI3K i EGFR i
OE
E
ACTA2 relative expression (Fold change)
F
Ctrl 1 OE 1 Ctrl 2 OE 2
pERK1/2 43 kDa
1 1.62 1.12 2.21
total ERK1/2 43 kDa
1 1.03 0.96 1.16
pERK/total ERK 1 1.57 1.17 1.91
Beta-Actin 42 kDa
G
Ctr
THBS1 antibody
Wound contraction
Time (days)
H
Control
THBS1 antibody
ns
I
Human RT- ex vivo wounds treated with THBS1 antibody (Real-time assessment)
D0 D2 D4 D6
Wound area change with time
Control
THBS1 antibody
Imaging with upright fluorescent microscope.
J
Human RT+ ex vivo wounds treated with siRUNX1 (Real-time assessment)
siCtr
siRUNX1
Wound area changed with time
K
Knockdown efficiency (%)
P = 0.0202
L
THBS1
Relative expression
P = 0.0429
M
FN1
P = 0.0023
N
Human RT- ex vivo wounds treated with siRUNX1 (Real-time assessment)

**Supplementary figure 9. Modulation of THBS1 or RUNX1 enhances the healing capacity of late irradiated human skin. (A)** CRISPR/dCas9 targeting THBS1 expression activation efficiency in fibroblast from healthy donors (*n* = 6). **(B)** Representative images and quantification of scratch wound assay in $RT^+$ fibroblasts transfected with siTHBS1 (*n* = 5). Scale bars: 300 µm. **(C)** GO analysis (right) showing differentially expressed (DE) genes (upregulated 45 genes and downregulated 138 genes) in RNA-seq of fibroblasts with THBS1 overexpression (OE, *n* = 2). qRT-PCR of ECM genes **(D)** and *ACTA2* **(E)** in fibroblasts with *THBS1* OE and treated with or not with signaling inhibitors (Control, *n* = 4; OE DMSO, *n* = 6; other groups, *n* = 3). **(F)** Western blotting of pEKR1/2, ERK, and beta-Actin in fibroblasts with *THBS1.* **(G)** Quantification of *ex vivo* wounds on $RT^-$ skin treated with or not with 66.7 µg/ml THBS1 antibodies (Ctr, *n* = 3; THBS1 antibody, *n* = 4). The treatment was topically applied on the wounds immediately after injury. Quantification **(H)** and representative images **(I)** of *ex vivo* wounds on $RT^-$ skin treated with or not with 0.2 µg/ml THBS1 antibodies (Control, *n* = 4; THBS1 antibody, *n* = 5). The treatment was topically applied on the wounds two days after injury. Wound contraction was evaluated by measuring the change of areas within the initial wound edges (indicated with dashed lines) on days 0-6 post-injury. Scale bars: 500 µm. Quantification and representative images of *ex vivo* wounds on $RT^+$ **(J)** or $RT^-$ **(N)** skin treated with or not with siRUNX1 (*n* = 3). Wound contraction was evaluated by measuring the change of areas within the initial wound edges on days 0-5 post-injury. Scale bars: 500 µm. **(K-M)** qRT-PCR of *RUNX1*, *THBS1*, and *FN1* in $RT^+$ *ex vivo* wound dermis treated with or not with siRUNX1 (siCtr, *n* = 7; siRUNX1, *n* = 6). Data are presented as means ± SD **(A, B, D, E, K-M)**, and means ± SEM **(G, H, J, N)**. Unpaired two-tailed student's t-test **(A, B, D, E, K-M)**, or two-way ANOVA **(G, H, J, N)**. ns, not significant ($p > 0.05$). Source data are provided as a Source Data file.

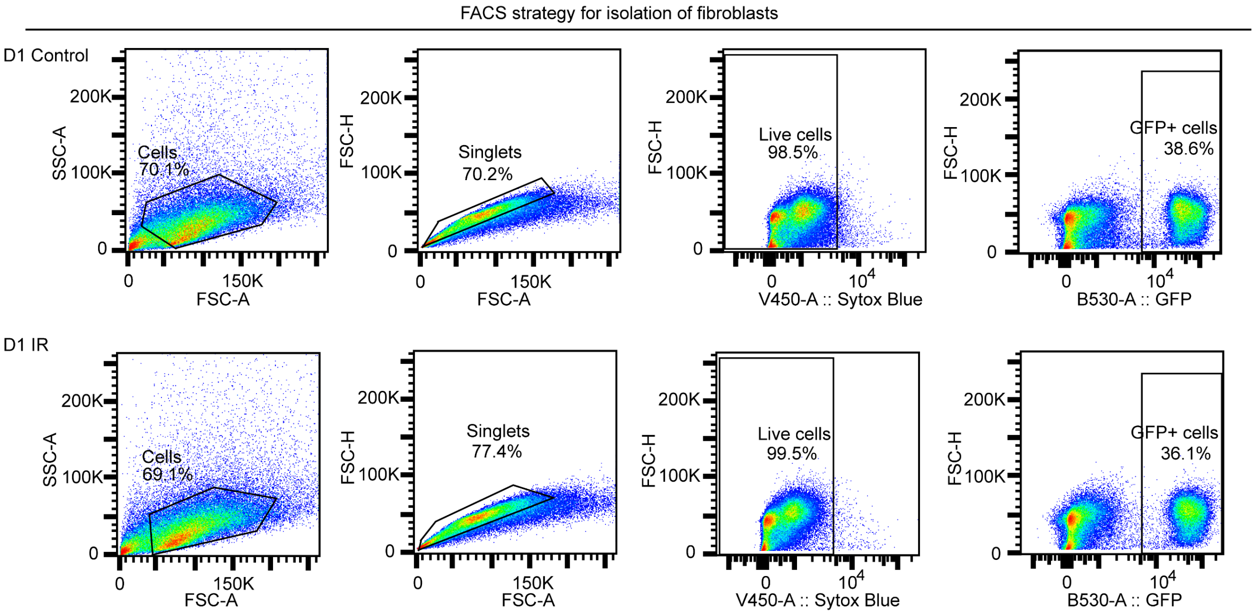


**Supplementary figure 10. FACS gating/sorting strategy for isolating fibroblasts from Pdgfra-H2Be-GFP murine skin.** Related to Supplementary figure 7I.

**Supplementary table 1. Characteristics of healthy donors involved in human *in vivo* wound healing model**

| Donor | Age (years) | Ethnicity | Experiment |
|---|---|---|---|
| 1 | 22-69 | Caucasian | RNA sequencing |
| 2 | | Caucasian | RNA sequencing |
| 3 | | Caucasian | RNA sequencing |
| 4 | | Caucasian | RNA sequencing |
| 5 | | Caucasian | RNA sequencing |
| 6 | | Caucasian | qRT-PCR |
| 7 | | Caucasian | qRT-PCR |
| 8 | | Caucasian | qRT-PCR |
| 9 | | Caucasian | qRT-PCR |
| 10 | | Caucasian | qRT-PCR |
| 11 | | Caucasian | Cell isolation, qRT-PCR, FISH |
| 12 | | Caucasian | Cell isolation, qRT-PCR |
| 13 | | Caucasian | Cell isolation, qRT-PCR |
| 14 | | Caucasian | Cell isolation, qRT-PCR |
| 15 | | Caucasian | Cell isolation, qRT-PCR |
| 16 | | Caucasian | ST, scRNA-seq |
| 17 | | Caucasian | scRNA-seq |
| 18 | | Caucasian | scRNA-seq |

ST: spatial transcriptomics.

**Supplementary table 2. HOMER motif analysis of the differentially accessible chromatin regions between RT$^{-}$ and RT$^{+}$ fibroblasts**

## RT$^{+}$ up domains

| Motif | TF match | p-value | # Target sequences with the motif | % of target sequences with the motif | # Background sequences with the motif | % of background sequences with the motif |
|---|---|---|---|---|---|---|
| 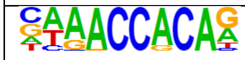 | RUNX | 1e-11 | 26.0 | 35.14 | 3290.9 | 6.86 |
| 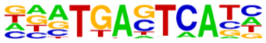 | Fra1 | 1e-3 | 22.0 | 29.73 | 2222.4 | 4.64 |
| 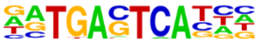 | Atf3 | 1e-11 | 24.0 | 32.43 | 2752.5 | 5.74 |
| 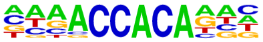 | RUNX2 | 1e-10 | 26.0 | 35.14 | 3968.0 | 8.28 |
| 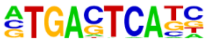 | AP-1 | 1e-10 | 24.0 | 32.43 | 3218.6 | 6.71 |
| 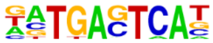 | BATF | 1e-10 | 23.0 | 31.08 | 2730.0 | 5.69 |
| 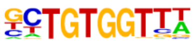 | RUNX | 1e-8 | 22.0 | 29.73 | 3386.5 | 7.06 |
| 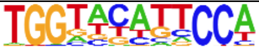 | FOSL2 | 1e-8 | 14.0 | 18.92 | 1223.8 | 2.55 |

## RT$^{-}$ up domains

| Motif | TF match | p-value | # Target sequences with the motif | % of target sequences with the motif | # Background sequences with the motif | % of background sequences with the motif |
|---|---|---|---|---|---|---|
| 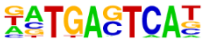 | BATF | 1e-23 | 41.0 | 42.27 | 2869.4 | 6.00 |
| 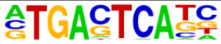 | AP-1 | 1e-22 | 42.0 | 43.30 | 3396.2 | 7.10 |
| 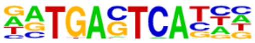 | Atf3 | 1e-21 | 39.0 | 40.21 | 2938.0 | 6.15 |
| 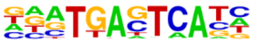 | Fra1 | 1e-19 | 34.0 | 35.05 | 2377.7 | 4.97 |
| 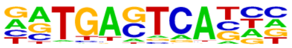 | Fosl2 | 1e-15 | 24.0 | 24.74 | 1388.3 | 2.90 |
| 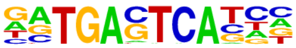 | Jun-AP1 | 1e-13 | 19.0 | 19.59 | 959.5 | 2.01 |
| 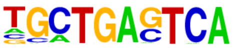 | Bach2 | 1e-7 | 2.0 | 12.37 | 799.7 | 1.67 |
| 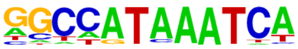 | Hoxc9 | 1e-5 | 16.0 | 16.49 | 2004.0 | 4.19 |

**Supplementary table 3. Gene Ontology analysis of the $RT^+$ up domain annotated genes that RUNX1 targets**

| Term | P-value | Genes |
|---|---|---|
| Regulation of myeloid cell differentiation (GO:0045637) | 1.58E-04 | **THBS1**; MEIS2; HOXA5 |
| Regulation of anatomical structure morphogenesis (GO:0022603) | 9.00E-04 | HECW2; KIF13B; EFNA5 |
| Regulation of cell migration (GO:0030334) | 0.00347258 | DACH1; GNA12; AMOTL2; **THBS1** |
| Negative regulation of transcription by RNA polymerase II (GO:0000122) | 0.003745099 | DACH1; ZNF608; SIN3B; SHOX2; MEIS2 |

**Supplementary table 4. List of sgRNAs and primers used in this study**

| Gene name | Sequence (5' ->3') / Cat.no |
|---|---|
| THBS1 | Hs.PT.58.45510824, IDT |
| CDKN1A | Hs.PT.58.40874346.g, IDT |
| ACTA2 | Hs.PT.56a.2542642, IDT |
| FN1 | Hs.PT.58.40005963, IDT |
| ELN | Hs.PT.58.4801194, IDT |
| COL1A1 | Hs.PT.58.15517795, IDT |
| COL3A1 | Hs.PT.58.4249241, IDT |
| RUNX1 | Hs.PT.58.2025564, IDT |
| PCNA | Hs.PT.58.24924035, IDT |
| Thbs1 | Mm.PT.58.11851002, IDT |
| Cdkn1a | Mm.PT.58.17125846, IDT |
| 18S | Forward: CGGCTACCACATCCAAGGAA<br>Reverse: GCTGTAATTACCGCGGCT<br>Probe: TGCTGGCACCAGACTTGCCCTC |
| GAPDH | Hs.PT.39a.22214836, IDT |
| Actb | Mm.PT.39a.22214843.g, IDT |
| Gapdh | Mm.PT.39a.1, IDT |
| ChIP-PCR RUNX1 motif 1 forward | TCTGTGACATGTGGTGATGGT |
| ChIP-PCR RUNX1 motif 1 reverse | GCGGACCTCACACTTACTG |
| ChIP-PCR RUNX1 motif 2 forward | TGCAGGAGTGTGTAAATAGACATGA |
| ChIP-PCR RUNX1 motif 2 reverse | CAGAGTCCAGCTGAAAGAGAGA |
| LKO.1 5' | GACTATCATATGCTTACCGT |
| gRNA_PCR_R | CAAGTTGATAACGGACTAGC |
| sgRNA 1 | GGCCGCCGCCCATTGGCCGG |
| sgRNA 2 | AGCGCCCCTTTAAAAGCGCG |
| sgRNA 3 | GGAGCCGCGCGCTTTTAAAG |
| sgRNA 4 | GCGGGAGGTGGGGGCCAGTC |
| sgRNA 5 | AAAGTGAAGGGGGCGGGGGT |
| sgRNA 6 | TAGCTGGAAAGTTGCGCGCC |
