# Supplementary material for: Epigenetic memory of radiotherapy in dermal fibroblasts impairs wound repair capacity in cancer survivors
Source: Nat Commun. 2024 Oct 28;15:9286. doi: 10.1038/s41467-024-53295-1 (PMC11519383; doi:10.1038/s41467-024-53295-1)
Supplement: Supplementary file 2 — Description of Additional Supplementary Files [file 41467_2024_53295_MOESM2_ESM.pdf]

Description of Additional Supplementary Files

File Name: Supplementary Data 1

Description: Information of breast cancer patients donating skin tissue samples.

File Name: Supplementary Data 2

Description: List of chromatin regions with differential accessibility in RT+ versus RT- fibroblasts.

File Name: Supplementary Data 3

Description: List of chromatin regions with differential accessibility in S+ versus S- fibroblasts.

File Name: Supplementary Data 4

Description: List of chromatin regions with IR-induced accessibility in fibroblasts.

File Name: Supplementary Data 5

Description: Gene Ontology analysis of differential accessibility domains annotated genes.

File Name: Supplementary Data 6

Description: List of differentially expressed genes in RT+ versus RT- fibroblasts.

File Name: Supplementary Data 7

Description: List of differentially expressed genes in RT- and RT+ fibroblasts treated with or not with TGFb.

File Name: Supplementary Data 8

Description: List of differentially expressed genes in human wounds (Day1 or Day7 vs. donor-match skin).

File Name: Supplementary Data 9

Description: List of chromatin regions with IR-induced accessibility in mouse fibroblasts (D1 IR vs Control) or mouse dermal cells (D7 IR vs Control).

File Name: Supplementary Data 10

Description: List of differentially expressed genes in human fibroblasts with THBS1 overexpression.
