# Supplementary material for: Epigenetic memory of radiotherapy in dermal fibroblasts impairs wound repair capacity in cancer survivors
Source: Nat Commun. 2024 Oct 28;15:9286. doi: 10.1038/s41467-024-53295-1 (PMC11519383; doi:10.1038/s41467-024-53295-1)
Supplement: Supplementary file 13 — Reporting Summary [file 41467_2024_53295_MOESM13_ESM.pdf]

nature portfolio

Corresponding author(s): DAPR NCOMMS-23-36416B

Last updated by author(s): Jul 24, 2024

Reporting Summary

Nature Portfolio wishes to improve the reproducibility of the work that we publish. This form provides structure for consistency and transparency in reporting. For further information on Nature Portfolio policies, see our Editorial Policies and the Editorial Policy Checklist.

Statistics

For all statistical analyses, confirm that the following items are present in the figure legend, table legend, main text, or Methods section.

n/a	Confirmed	
☐	☒	The exact sample size (*n*) for each experimental group/condition, given as a discrete number and unit of measurement
☐	☒	A statement on whether measurements were taken from distinct samples or whether the same sample was measured repeatedly
☐	☒	The statistical test(s) used AND whether they are one- or two-sided<br>*Only common tests should be described solely by name; describe more complex techniques in the Methods section.*
☐	☒	A description of all covariates tested
☐	☒	A description of any assumptions or corrections, such as tests of normality and adjustment for multiple comparisons
☐	☒	A full description of the statistical parameters including central tendency (e.g. means) or other basic estimates (e.g. regression coefficient) AND variation (e.g. standard deviation) or associated estimates of uncertainty (e.g. confidence intervals)
☐	☒	For null hypothesis testing, the test statistic (e.g. *F*, *t*, *r*) with confidence intervals, effect sizes, degrees of freedom and *P* value noted<br>*Give P values as exact values whenever suitable.*
☒	☐	For Bayesian analysis, information on the choice of priors and Markov chain Monte Carlo settings
☒	☐	For hierarchical and complex designs, identification of the appropriate level for tests and full reporting of outcomes
☐	☒	Estimates of effect sizes (e.g. Cohen's *d*, Pearson's *r*), indicating how they were calculated

Our web collection on statistics for biologists contains articles on many of the points above.

Software and code

Policy information about availability of computer code

Data collection	qRT-PCR data were collected by QuantStudio 6 or 7 (Applied Biosystems, Waltham, MA).<br>Immunoflorescence staining images were acquired using Zeiss LSM900-Airy confocal or Zeiss AxioScan.Z1 Slide Scanner (Zeiss, Germany).<br>Masson's trichrome staining images were acquired using Nikon eclipse Ni-E fluorescence microscope (Nikon, Japan).<br>Cell proliferation and scratch wound assay images were acquired using IncuCyte ZOOM imaging system (Sartorius, Germany).<br>Sequencing data were acquired using Novaseq 6000, X, X Plus sequencer (Illumina, California).<br>Western blot developed images were acquired using ChemiDocTM MP Imaging System. (Bio-Rad, California).
Data analysis	Statistic analysis were used GraphPad Prism version 9. Microscope images quantification was used ImageJ version 1.53 k and Zen 3.4 software. Bioinformatic analysis were used Metascape (v3.5.20240101), TOBIAS software (v0.14.0), R software (v4.3.3), HOMER motif analysis (v4.11), Cell Ranger (v5.0.1), Space Ranger (v1.2), Seurat (v4.1.0), CellChat (v1.4.0), MACS2 (v2.2.6), Trimmomatic (v0.36), Bowtie2 (v2.3.5.1), deepTools (v3.3.2), BEDTools (v2.29.2), DESeq2 (v1.44.0), ChIPseeker (v1.40.0), Scrublet (v0.2.3), DoubletFinder (v2.0.4) and SCTransform (v0.4.1).

For manuscripts utilizing custom algorithms or software that are central to the research but not yet described in published literature, software must be made available to editors and reviewers. We strongly encourage code deposition in a community repository (e.g. GitHub). See the Nature Portfolio guidelines for submitting code & software for further information.

Data

Policy information about availability of data

All manuscripts must include a data availability statement. This statement should provide the following information, where applicable:

- Accession codes, unique identifiers, or web links for publicly available datasets
- A description of any restrictions on data availability
- For clinical datasets or third party data, please ensure that the statement adheres to our policy

The raw and processed sequencing data generated in this study have been deposited in the Gene Expression Omnibus (GEO) database: ATAC-seq data (GSE254753), RNA-seq data (GSE254756), and single cell multiome ATAC+RNA-seq data (GSE254758). Bulk RNA-seq data (human in vivo wound healing) was published previously and can be accessed via GSE174661. Source data are provided with this paper. Any additional data that support the findings of this study are available from the corresponding author upon request.

Research involving human participants, their data, or biological material

Policy information about studies with human participants or human data. See also policy information about sex, gender (identity/presentation), and sexual orientation and race, ethnicity and racism.

Reporting on sex and gender	Sex were not considered in the study design. All skin samples were collected from female breast cancer patients during reconstruction surgery (human irradiated and non-irradiated skin samples).
Reporting on race, ethnicity, or other socially relevant groupings	Race, ethnicity or socially relevant groupings were not considered in study design.
Population characteristics	The information of breast cancer patients donating skin tissue samples was listed in the supplementary table S1. The information of healthy donors involved in human in vivo wound healing model was listed in supplementary table S2. All skin samples were collected from female breast cancer patients aged 33-67 years. Human skin and wound samples were collected from both female and male participants aged 22-69 years.
Recruitment	Breast cancer patients who had previously undergone external beam radiotherapy and mastectomy were recruited for this study. During their breast reconstruction surgery, we collected both irradiated and non-irradiated skin samples. We also recruited healthy volunteers, with no significant health issues, to participate in our human in vivo wound healing model. We do not have access to the personal information of the donors, so we cannot determine if there is a self-selection bias in the data. It's possible that different segments of the public have varying propensities to donate to scientific research. However, we do not anticipate this significantly impacting the study's results.
Ethics oversight	Research involving human subjects was approved by the Stockholm local ethics committee with appropriate informed consent.

Note that full information on the approval of the study protocol must also be provided in the manuscript.

Field-specific reporting

Please select the one below that is the best fit for your research. If you are not sure, read the appropriate sections before making your selection.

☒ Life sciences ☐ Behavioural & social sciences ☐ Ecological, evolutionary & environmental sciences

For a reference copy of the document with all sections, see nature.com/documents/nr-reporting-summary-flat.pdf

Life sciences study design

All studies must disclose on these points even when the disclosure is negative.

Sample size	No power calculations were performed to determine sample size. Sample size was dictated by the availability of scarce clinical samples, and based on prior experience with similar studies.
Data exclusions	Only the data from samples determined to have poor cell/tissue viability or poor RNA quality were excluded.
Replication	At least three biological replicates were performed for all the experiments unless otherwise stated in the figure legends or Methods. All attempts at replication were successful.
Randomization	For in vitro studies, conditions were randomly assigned to each experimental treatment. For in vivo study, the mice were randomly assigned to each group.
Blinding	The investigators were not blinded to group allocation during data collection and/or analysis, as this was an exploratory study with anonymous untreated samples.

Reporting for specific materials, systems and methods

We require information from authors about some types of materials, experimental systems and methods used in many studies. Here, indicate whether each material, system or method listed is relevant to your study. If you are not sure if a list item applies to your research, read the appropriate section before selecting a response.

Materials & experimental systems

n/a	Involved in the study
☐	☒ Antibodies
☒	☐ Eukaryotic cell lines
☒	☐ Palaeontology and archaeology
☐	☒ Animals and other organisms
☒	☐ Clinical data
☒	☐ Dual use research of concern
☒	☐ Plants

Methods

n/a	Involved in the study
☒	☐ ChIP-seq
☒	☐ Flow cytometry
☒	☐ MRI-based neuroimaging

Antibodies

Antibodies used

Mouse monoclonal anti-human THBS1 protein, Santa Cruz Biotechnology, Cat#sc-59887, Clone A6.1; 1:100 dilution for IF staining.
Mouse monoclonal anti-human THBS1 protein, Invitrogen, Cat#MS5-13377, Clone A4.1; 66.7 μg/mL or 0.2 μg/mL per biopsy for ex vivo wound healing experiment.
Rabbit monoclonal anti-human RUNX1, Abcam, Cat#ab272456, Clone EPR23309-113; 2.5 μg per reaction for ChIP-PCR.
Rabbit polyclonal anti-Histone H3 (mono methyl K4), Abcam, Cat#ab8895; 2 μg per reaction for ChIP-PCR.
Anti-Thrombospondin 1 antibody [EPR22927-54], Abcam, Cat#ab267388; 1:100 dilution for IF staining.
Mouse PDGF R alpha Antibody, R&D, Cat#AF1062. 1:15 dilution for IF staining, 1:10 dilution for RNA in situ and protein co-staining,
Phospho-SMAD2 (Ser465, Ser467) Polyclonal Antibody, Thermofisher, Cat#44-244G. 1:100 dilution for IF staining.

Validation

Each primary antibody was validated by manufacturer's product page.
Mouse monoclonal anti-human THBS1 protein, Santa Cruz Biotechnology, Cat#sc-59887, Clone A6.1
https://www.scbt.com/p/thrombospondin-1-antibody-a6-1?requestFrom=search

Mouse monoclonal anti-human THBS1 protein, Invitrogen, Cat#MS5-13377, Clone A4.1
https://www.thermofisher.com/antibody/product/Thrombospondin-1-Antibody-clone-A4-1-Monoclonal/MA5-13377

Rabbit monoclonal anti-human RUNX1, Abcam, Cat#ab272456, Clone EPR23309-113
https://www.abcam.com/products/primary-antibodies/runx1--aml1-antibody-epr23309-113-chip-grade-ab272456.html

Rabbit polyclonal anti-Histone H3 (mono methyl K4), Abcam, Cat#ab8895
https://www.abcam.com/products/primary-antibodies/histone-h3-mono-methyl-k4-antibody-chip-grade-ab8895.html?productWallTab=ShowAll

Anti-Thrombospondin 1 antibody [EPR22927-54], Abcam, Cat#ab267388
https://www.abcam.com/en-se/products/primary-antibodies/thrombospondin-1-antibody-epr22927-54-ab267388#

Mouse PDGF R alpha Antibody, R&D, Cat#AF1062;
https://www.rndsystems.com/products/mouse-pdgf-ralpha-antibody_af1062

Phospho-SMAD2 (Ser465, Ser467) Polyclonal Antibody, Thermofisher, Cat#44-244G
https://www.thermofisher.com/antibody/product/Phospho-SMAD2-Ser465-Ser467-Antibody-Polyclonal/44-244G

Animals and other research organisms

Policy information about studies involving animals; ARRIVE guidelines recommended for reporting animal research, and Sex and Gender in Research

Laboratory animals

CD-1 mice, wild-type C57BL/6 mice, and Pdgfra-H2BeGFP mouse line were used in this study. Male, age 8-12 weeks at start of the experiment.

Wild animals

No wild animals were used in the study.

Reporting on sex

Male mice was used in this study.

Field-collected samples

The study did not involve samples collected from field.

Ethics oversight

The protocols (#17-051 and #11854-2020) of murine experiments were approved by the Comité de Protection des Animaux de

Ethics oversight	l'Université Laval (CPAUL), Cégep de Sainte-Foy Animal Protection Committee (Québec, Canada), and the North Stockholm Ethical Committee for Care and Use of Laboratory Animals (Stockholm, Sweden).

Note that full information on the approval of the study protocol must also be provided in the manuscript.

Plants

Seed stocks	N/A
Novel plant genotypes	N/A
Authentication	N/A
